# Altered senescence and mitochondrial transcriptome defines age-related changes in satellite cells

**DOI:** 10.3389/fcell.2025.1699206

**Published:** 2026-01-27

**Authors:** Fasih A. Rahman, Joe Quadrilatero

**Affiliations:** Department of Kinesiology and Health Sciences, University of Waterloo, Waterloo, ON, Canada

**Keywords:** skeletal muscle, satellite cell, skeletal muscle regeneration, aging, senescence, SASP, mitochondrial remodeling

## Abstract

Aging impairs the regenerative capacity of skeletal muscle in part through the functional decline of the resident stem cell population called satellite cells. With age, satellite cells exhibit a loss of quiescence, altered proliferation, and impaired differentiation, leading to incomplete myogenesis following injury. Mitochondria are central to stem cell function, providing ATP, regulating redox homeostasis, and integrating several signaling pathways during lineage progression. While mitochondrial remodeling and function is essential for supporting the metabolic demands of myogenesis, the extent to which these processes are altered in aged satellite cells across cell states remains unclear. To address this, we performed a comparative transcriptomic analysis of young and aged satellite cells in quiescent, proliferating, and early differentiating states using three publicly available microarray datasets. Our results reveal that aged satellite cells exhibit a dysregulated senescence profile, characterized by the simultaneous upregulation of both senescence-inducing and -inhibiting genes, suggestive of a metastable senescence state. These features persisted during early differentiation, where aged cells also displayed increased expression of senescence-associated secretory phenotype (SASP) components, potentially contributing to a pro-inflammatory niche. Mitochondrial gene expression was relatively stable in quiescent cells but showed marked remodeling upon activation, particularly in aged cells. While young satellite cells upregulated transcriptional programs related to mitochondrial function, aged cells exhibited broader and less coordinated responses enriched for stress, apoptotic, and metabolic pathways. Despite evidence of mitochondrial stress, mitophagy gene activation remained limited in aged cells, raising the possibility of impaired organelle quality control. Together, our findings highlight age-associated disruptions in both senescence and mitochondrial remodeling programs across the satellite cell lifecycle. These transcriptional changes likely underlie impaired regenerative responses in aging muscle and identify potential targets for rejuvenating muscle stem cell function.

## Introduction

Aging is accompanied by a progressive and multifactorial decline in the function of virtually all physiological systems, contributing to increased frailty, disease burden, and reduced regenerative capacity in older individuals (López-Otín et al., 2013; Dodig et al., 2019; Tenchov et al., 2023). While this decline reflects the combined effects of genomic instability, proteostatic stress, metabolic alterations, and chronic low-grade inflammation, a critical component of age-associated tissue deterioration is the loss of stem cell function (López-Otín et al., 2013; Dodig et al., 2019; Tenchov et al., 2023). Adult stem cell populations are essential for tissue maintenance and regeneration throughout life, replenishing differentiated cells during homeostasis and responding to injury with rapid expansion and lineage-specific differentiation (Hawke and Garry, 2001; Dumont et al., 2015b; Dumont et al., 2015a). With advancing age, these regenerative cell populations undergo stem cell exhaustion, characterized by diminished self-renewal, reduced proliferative potential, and altered lineage commitment (Price et al., 2014; Tierney et al., 2014; García-Prat et al., 2020). These changes ultimately limit the ability of tissues to regenerate and adapt to physiological stressors. In skeletal muscle, the classic example of this phenomenon is the functional deterioration of the resident skeletal muscle stem cells (i.e., satellite cells) responsible for postnatal growth and regeneration (Seale and Rudnicki, 2000; Seale et al., 2000; Oustanina et al., 2004; Kuang et al., 2007; Lepper et al., 2011; Murphy et al., 2011). Satellite cells are maintained in a reversible quiescent state under basal conditions (Hawke and Garry, 2001; Dumont et al., 2015b; Dumont et al., 2015a). Following skeletal muscle injury, they are activated, re-enter the cell cycle, proliferate, differentiate, and fuse with damaged or newly forming fibers to restore tissue integrity (Hawke and Garry, 2001; Dumont et al., 2015b; Dumont et al., 2015a). However, in aged skeletal muscle, satellite cells exhibit profound functional impairments. They display delayed activation, reduced proliferation rates, and an impaired ability to fully engage in the myogenic differentiation program, often stalling or deviating toward maladaptive fates, including cell death (Alway et al., 2014; Brack and Muñoz-Cánoves, 2016; Liu et al., 2018; Muñoz-Cánoves et al., 2020; Rahman and Quadrilatero, 2023; Haroon et al., 2022; Rahman et al., 2024). Moreover, these deficits result in a markedly diminished regenerative response in aged skeletal muscle, particularly after acute or catastrophic skeletal muscle injuries. Studies using transplantation and heterochronic parabiosis have demonstrated that both intrinsic alterations within aged satellite cells and extrinsic changes in the aged niche contribute to this decline (Carlson and Faulkner, 1989; Conboy and Rando, 2005; Brack and Rando, 2007; Conboy et al., 2013; Sousa-Victor et al., 2014a; Brack and Muñoz-Cánoves, 2016). Our previous work and others have shown that *in vitro* models of C2C12 myoblast aging, such as sequential passaging, exhibit heightened stress responses and increased mitochondrial apoptotic signaling (Sharples et al., 2011; Shahini et al., 2018; Shahini et al., 2021; Rahman et al., 2024). Importantly, these deficits coincide with disrupted mitochondrial function, suggesting that mitochondrial health is a key determinant of stem cell capacity.

Mitochondria are central regulators of stem cell fate, not only by supplying ATP to meet the energetic demands of proliferation and differentiation, but also by integrating biosynthetic pathways, maintaining redox balance, and modulating signaling networks (Wanet et al., 2015; Robles-Flores et al., 2021; Hong et al., 2022; Li et al., 2023; Garone et al., 2024). During myogenic differentiation, mitochondrial remodeling is critical for the transition from a low-energy state in quiescence to a higher energy demanding state that supports the bioenergetic demands of lineage progression (Vertel and Fischman, 1977; Barbieri et al., 2011; Latil et al., 2012; Robinson et al., 2019; Rahman and Quadrilatero, 2023). This remodeling involves coordinated mitochondrial biogenesis, changes in morphology through fission and fusion, and quality control *via* mitophagy (Sin et al., 2016; Baechler et al., 2019; Rahman and Quadrilatero, 2021; Quadrilatero, 2023; Rahman et al., 2025a). Conversely, mitochondrial dysfunction, characterized by reduced respiratory capacity, elevated reactive oxygen species (ROS) production, and defective mitophagy, can impair these processes, triggering stress responses such as apoptosis or senescence, and ultimately contributing to the decline in satellite cell function with age (García-Prat et al., 2016; Rahman et al., 2024). A growing body of evidence suggests that features of cellular senescence, including stable cell cycle arrest, a senescence-associated secretory phenotype (SASP), and altered metabolic regulation, become more evident in aged satellite cells. These senescence-like changes may exacerbate mitochondrial stress while promoting a chronic inflammatory environment that further impairs regeneration (He et al., 2021; Moiseeva et al., 2023; Wang et al., 2025). However, the specific transcriptional remodeling of mitochondrial pathways across different activation states in young versus aged satellite cells remains poorly defined.

In the present study, we address this gap by performing a comparative analysis of senescence and mitochondrial gene expression programs in quiescent, proliferating, and differentiating satellite cells from young and aged skeletal muscle. Using publicly available microarray datasets (GSE50821, GSE273 and GSE47177), we systematically examine age-dependent differences in senescence and mitochondrial transcriptional profiles in each state. By investigating these datasets, we aim to determine whether mitochondrial remodeling in aged satellite cells represents an adaptive response to cellular stress or a maladaptive process that compromises myogenesis and thus impairs skeletal muscle regeneration. Moreover, our work provides insight into the mitochondrial mechanisms underlying age-related decline in satellite cell function and identifies potential targets for strategies aimed at preserving or restoring skeletal muscle regenerative capacity in aging muscle.

## Methods

### Data acquisition

Publicly available transcriptomic datasets were retrieved from the NCBI Gene Expression Omnibus (GEO) database using keywords such as “satellite cell”, “skeletal muscle”, “aging”, and “differentiation”. Datasets were included if they met the following criteria: (1) derived from mouse, (2) contained at least two biological replicates per condition, and (3) utilized sequencing platforms with accessible raw or normalized count data. From this search, we found three datasets for analysis (GSE50821, GSE273, and GSE47177). GSE50821 employed fluorescence-activated cell sorting (FACS) using a defined surface marker profile (CD45^−^ Sca-1^-^ CD11b^−^ CXCR4^+^ β1-integrin^+^), which distinguishes quiescent satellite cells from non-myogenic and more differentiated muscle populations (Sinha et al., 2014). GSE273 used a pre-plating method to isolate satellite cells, yielding a population reported to be >99% PAX7^+^ and DES^+^ (Beggs et al., 2004). In this dataset, the authors pooled four samples per group to reduce experimental variability (Beggs et al., 2004). Based on the pre-plate isolation method, culture conditions, and stable expression of PAX7 and DES, these cells are in a proliferative state (Seale et al., 2000; Pawlikowski et al., 2009). GSE47177 also used FACS to isolate satellite cells, applying the marker combination VCAM1^+^ CD31^−^ CD45^−^ Sca-1^-^ to isolate satellite cells from uninjured skeletal muscle and 60 h following BaCl_2_-induced injury in young and aged mice (Liu et al., 2013). At this timepoint, satellite cells are transitioning into an early differentiation state, supported by studies showing upregulated MYOG expression 2 days post-injury (Borok et al., 2021). Summary of datasets can be found in Table 1.

**TABLE 1:** Metadata summary for transcriptomic datasets.

GEO accession	References	Biological context/cell state	Isolation method	Mouse strain	Sex	Age (young/aged)	Injury/culture condition	Platform	Replicates per group
GSE50821	Sinha et al. (2014)	Quiescent satellite cells (freshly isolated)	FACS (CD45^−^ Sca-1^-^ CD11b^−^ CXCR4^+^ β1-integrin^+^)	C57BL/6	Male	2 months/24 months	Uninjured	Affymetrix mouse genome 430 2.0 array	5
GSE273	Beggs et al. (2004)	Proliferating myogenic cells (pre-plating culture)	Pre-plate enrichment (>99% PAX7^+^ DES^+^)	DBA/2JNIA	Female	8 months/23 months	Pre-plate 3 days post-freeze injury	Affymetrix murine genome U74A version 2 array	2 (pooled samples)
GSE47177	Liu et al. (2013)	Early differentiating satellite cells (60 h post-injury)	FACS (VCAM1^+^ CD31^−^ CD45^−^ Sca-1^-^)	Unknown	Unknown	2 months/24 months	Uninjured or 60 h post-BaCl_2_ injury	Affymetrix mouse gene 1.0 ST array	3

### Differential gene expression analysis

For count-based datasets analyzed with DESeq2 (GSE50821, GSE273), raw count matrices were imported into R (version 4.5.1) and inspected for non-finite or negative values. Any negative entries were set to zero and counts were coerced to integers (rounding) prior to model fitting. Genes were filtered to retain those with counts >10 in at least 2 samples per group. Library size normalization was performed using DESeq2’s median-of-ratios method, and dispersion was estimated with the Wald test using a positive-count size factor type. The “Young” group was set as the reference level, and contrasts were computed as “Aged” vs. “Young” within each state. Default independent filtering and Benjamini–Hochberg false discovery rate (FDR) control was used. For intensity-based microarray data analyzed with limma (GSE47177), we used pre-normalized, log_2_-transformed expression matrices obtained directly from GEO. Because raw files were not available, we retained the authors’ original normalization and quality-control procedures as described in the associated dataset record. These matrices were used as input for linear modeling with limma. Analyses were stratified to match the specific biological contrast used (e.g., Activated vs. Quiescent for each group). A linear model was fit and moderated t-statistics were obtained via empirical Bayes shrinkage. Multiple testing was controlled using the Benjamini–Hochberg FDR. Probe identifiers were mapped to official MGI gene symbols using platform annotations. Genes were defined as differentially expressed if they met an adjusted p-value (FDR) < 0.05 and an absolute log_2_ fold-change ≥1. Differentially expressed gene lists were subsequently used as input for pathway and enrichment analyses described below. For downstream visualization (volcano plots, heatmaps), z-scores were computed from variance-stabilized counts (DESeq2) or from the pre-normalized log_2_ intensities (limma).

### Functional enrichment and pathway analysis

Principal Component Analysis (PCA) was performed on variance-stabilized transformed (VST) counts using the vst function from the DESeq2 package or performed on log_2_-transformed, quantile-normalized expression values using the prcomp function in R. Gene Ontology (GO) and pathway enrichment analyses were performed using the clusterProfiler and enrichR packages. Genes were categorized as upregulated if Log2FC > 0.5 and downregulated if Log2FC < −0.5 irrespective of adjusted p-value, to capture broader directional transcriptional trends. Enrichment analyses were conducted using KEGG and Reactome gene sets. To minimize redundancy in GO terms, semantic simplification was performed using REVIGO (Supek et al., 2011). Pathway-level visualization was additionally conducted using Pathview.

### Integration with CellAge and MitoCarta databases

To further characterize age-related and mitochondrial transcriptional changes, differentially expressed genes (DEGs) were cross-referenced with the CellAge Build 3 database (Avelar et al., 2020), which catalogues genes associated with cellular senescence, and with the MitoCarta 3.0 database (Rath et al., 2021), which identifies nuclear-encoded genes with known mitochondrial localization. Overlaps between DEGs and these databases were extracted to identify senescence-related and mitochondria-associated genes that were differentially expressed across conditions. DEGs were categorized into functional groups derived from MitoCarta 3.0, including autophagy, apoptosis, mitochondrial dynamics (i.e., fission and fusion), oxidative phosphorylation (OXPHOS), antioxidant function, lipid metabolism, and carbohydrate metabolism. Functional grouping of genes was based on curated annotation categories provided in MitoCarta 3.0. These subsets were used to interrogate the relationship between cellular aging and mitochondrial gene expression, specifically within quiescent, proliferating, and differentiating satellite cell populations from young and aged muscle.

### Data visualization

Volcano plots, bar graphs, and heatmaps were generated using exported z-score or normalized expression data from the DEG analyses and visualized in GraphPad Prism. Reactome-based ridge plots were generated using the ReactomePA package. Semantic similarity plots from GO analysis were created using REVIGO in R (version 4.5.1). Figures were assembled to highlight key transcriptional changes related to mitochondrial function and cellular senescence.

## Results

### Distinct senescence and mitochondrial signatures define aged quiescent satellite cells

**FIGURE 1:**
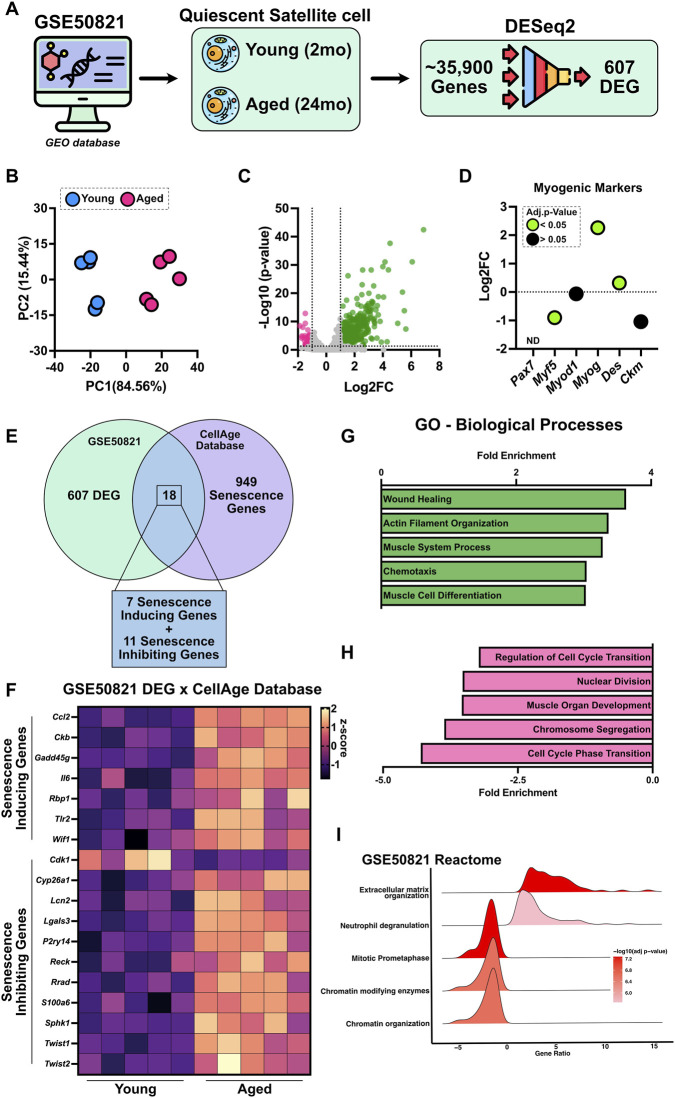
Aging alters transcriptomic and senescence-associated programs in quiescent satellite cells (GSE50821). **(A)** Schematic overview of the analysis pipeline for dataset GSE50821, which profiled isolated quiescent satellite cells from young (2-month-old) and aged (24-month-old) mice. Differential expression analysis using DESeq2 identified 607 significantly altered genes between groups. **(B)** Principal component analysis (PCA) of variance-stabilized gene expression data shows clear separation between young and aged cells, with PC1 and PC2 explaining 84.56% and 15.44% of the total variance, respectively. **(C)** Volcano plot depicting differentially expressed genes (upregulated in green and downregulated in pink). **(D)** Expression profiles of canonical myogenic markers (*Pax7*, *Myf5*, *Myod1*, *Myog*, *Des*, and *Ckm*) between aged and young quiescent satellite cells. Data are shown as Log2FC (aged relative to young), where positive values indicate higher expression in aged cells. Green points denote genes with an adjusted p-value <0.05, while black points represent non-significant changes. **(E)** Venn diagram showing the overlap between GSE50821 DEGs and the CellAge Build 3 senescence gene database, identifying 18 senescence-associated genes. **(F)** Heatmap displaying the expression patterns of the 18 overlapping senescence-inducing and -inhibiting genes. **(G)** Gene ontology (GO) enrichment analysis of biological processes reveals upregulation (green) of stress signaling and skeletal muscle remodeling pathways, and **(H)** downregulation (pink) of pathways related to cell cycle regulation. **(I)** Reactome pathway analysis revealed increased enrichment of extracellular matrix organization pathways and a reduction in genomic maintenance pathways in aged quiescent satellite cells.

To investigate how aging alters the baseline transcriptional landscape of skeletal muscle stem cells, we first examined gene expression changes in quiescent satellite cells from young and aged mice. We analyzed GSE50821, which profiled FACS-isolated quiescent satellite cells from young (2-month-old) and aged (24-month-old) mice (Figure 1A). PCA revealed clear separation between young and aged samples, with PC1 accounting for 84.56% and PC2 for 15.44% of the total variance (Figure 1B), indicating robust age-associated transcriptomic divergence (Figure 1B). Of the 35,900 genes analyzed, 607 were identified as differentially expressed between young and aged satellite cells (Figure 1C). In total, 524 genes were upregulated and 83 genes were downregulated in aged cells. We examined the expression of canonical myogenic markers (*Pax7*, *Myf5*, *Myod1*, *Myog*, *Des*, and *Ckm*; Figure 1D). Although *Pax7* expression was undetectable, aged quiescent cells showed lower *Myf5* expression, but higher *Myog* and *Des* expression compared to young cells, suggesting an early expression of differentiation-associated genes. Nonetheless, when these DEGs were cross-referenced with the CellAge Build 3 database, 18 senescence-associated genes were identified (Figure 1E). Notably, all 7 senescence-inducing genes were upregulated (*Ccl2*, *Ckb*, *Gadd45g*, *Il6*, *Rbp1*, *Tlr2*, *Wif1*), while only one senescence-inhibiting gene was downregulated (*Cdk1*) and the remaining 10 senescence-inhibiting genes were unexpectedly upregulated (*Cyp26a1*, *Lcn2*, *Lgals3*, *P2ry14*, *Reck*, *Rrad*, *S100a6*, *Sphk1*, *Twist1*, *Twist2*; Figure 1F). GO analysis of the DEGs revealed significant enrichment of pathways associated with wound healing, actin filament organization, muscle system processes, chemotaxis, and muscle cell differentiation (Figure 1G). In contrast, downregulated GO terms included processes critical for cell cycle progression, genomic stability, and muscle organ development (Figure 1H). Reactome pathway analysis reinforced these observations, highlighting upregulation of genes involved in extracellular matrix remodeling and neutrophil degranulation, while pathways related to mitotic prometaphase, chromatin-modifying enzyme activity, and chromatin organization were notably suppressed (Figure 1I). Collectively, these findings suggest that aged quiescent satellite cells adopt a transcriptional profile characterized by enhanced cytoskeletal and matrix remodeling activity, coupled with diminished proliferative potential and chromatin regulatory capacity.

**FIGURE 2:**
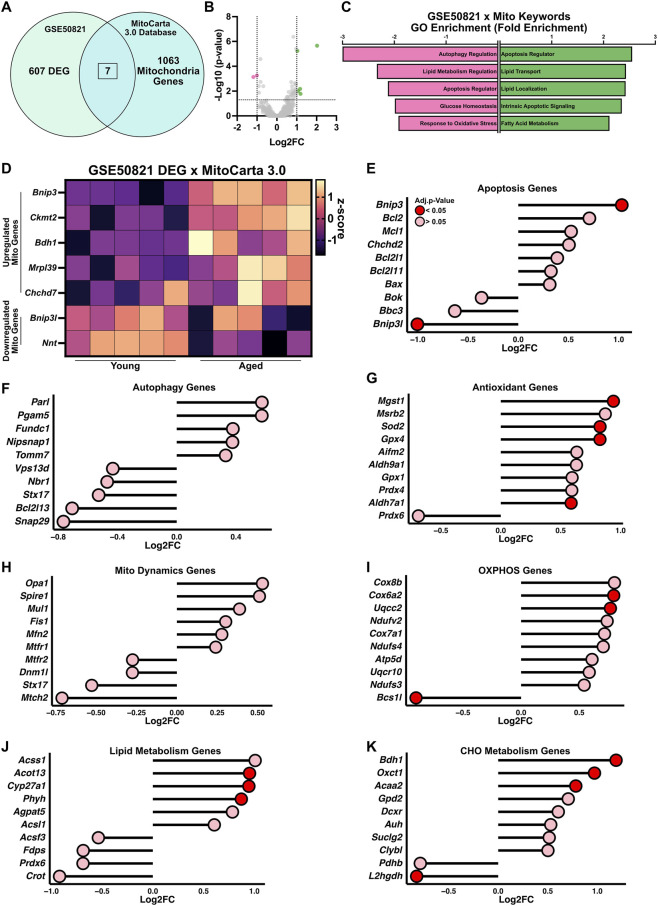
Mitochondrial and metabolic gene expression changes in aged quiescent satellite cells (GSE50821). **(A)** Differentially expressed genes (DEGs) from GSE50821 were cross-referenced with the MitoCarta 3.0 database to identify 7 mitochondrial-associated DEGs in aged quiescent satellite cells. **(B)** Volcano plot highlighting mitochondrial DEGs (upregulated in green and downregulated in pink). **(C)** Gene ontology (GO) enrichment analysis of mitochondrial DEGs shows distinct pathway enrichment among upregulated (green) and downregulated (pink) genes. **(D)** Heatmap displaying expression patterns of the 7 mitochondrial DEGs. Lollipop plots of selected mitochondrial-related genes, categorized into seven functional categories: **(E)** apoptosis, **(F)** autophagy, **(G)** antioxidant, **(H)** mitochondrial dynamics, **(I)** oxidative phosphorylation (OXPHOS), **(J)** lipid metabolism, and **(K)** carbohydrate (CHO) metabolism.

To determine whether mitochondrial programs are disrupted in aged quiescent satellite cells, we cross-referenced the 607 differentially expressed genes with the MitoCarta 3.0 database (Figure 2A). This analysis identified 7 mitochondrial-associated genes that were differentially expressed with age, including five upregulated and two downregulated transcripts (Figure 2B). GO analysis of the upregulated mitochondrial genes revealed enrichment in pathways related to apoptotic signaling, lipid transport, lipid localization, and fatty acid metabolism (Figure 2C). In contrast, the downregulated mitochondrial genes were associated with essential processes such as autophagy regulation, lipid metabolism regulation, apoptotic regulation, glucose homeostasis, and the cellular response to oxidative stress.

To further evaluate the functional relevance of the transcriptomic changes observed in aged quiescent satellite cells, we categorized all genes overlapping with the MitoCarta database into seven key mitochondrial and stress-related functional categories including apoptosis, autophagy, mitochondrial dynamics, antioxidant, OXPHOS, lipid metabolism, and carbohydrate metabolism. In the apoptosis category, aged cells showed upregulation of *Bnip3* (Log2FC = 1) but a downregulation of *Bnip3l* (Log2FC = −1) (Figures 2D–F); importantly, both genes also serve as mitophagy receptors, linking apoptotic signaling with mitochondrial quality control. No autophagy-related genes were differentially expressed, suggesting minimal transcriptional regulation of canonical autophagy pathways in the quiescent state. Similarly, genes associated with mitochondrial dynamics remained unchanged (Figure 2G), indicating that large-scale remodeling of mitochondrial morphology is likely not initiated until satellite cells are activated. In the antioxidant gene category, aged cells displayed upregulation of *Mgst1* (Log2FC = 0.94), *Sod2* (Log2FC = 0.83), *Gpx4* (Log2FC = 0.82), and *Aldh7a1* (Log2FC = 0.58), possibly reflecting an enhanced need for detoxification of reactive byproducts or a compensatory adaptation to oxidative stress (Figure 2H). Within the OXPHOS category, several genes encoding components of the mitochondrial respiratory chain were upregulated, including *Cox6a2* (Log2FC = 0.8) and *Uqcc2* (Log2FC = 0.76), while *Bcs1l* (Log2FC = −0.9), a gene involved in respiratory complex III assembly, was downregulated (Figure 2I). In the lipid metabolism category, aged satellite cells exhibited upregulation of *Acot13* (Log2FC = 0.95), *Cyp27a1* (Log2FC = 0.95), and *Phyh* (Log2FC = 0.87), consistent with a shift toward increased lipid utilization or remodeling (Figure 2J). Finally, in the carbohydrate metabolism category, genes such as *Bdh1* (Log2FC = 1.2), *Oxct1* (Log2FC = 0.97), and *Acaa2* (Log2FC = 0.78) were upregulated, whereas *L2hgdh* (Log2FC = −0.82) was downregulated (Figure 2K). Collectively, these results highlight a metabolic rewiring in aged quiescent satellite cells, characterized by modest but functionally relevant changes in mitochondrial gene expression that may compromise bioenergetic flexibility and redox balance prior to activation.

### Proliferating satellite cells exhibit coordinated shifts in senescence and mitochondrial transcriptome

**FIGURE 3:**
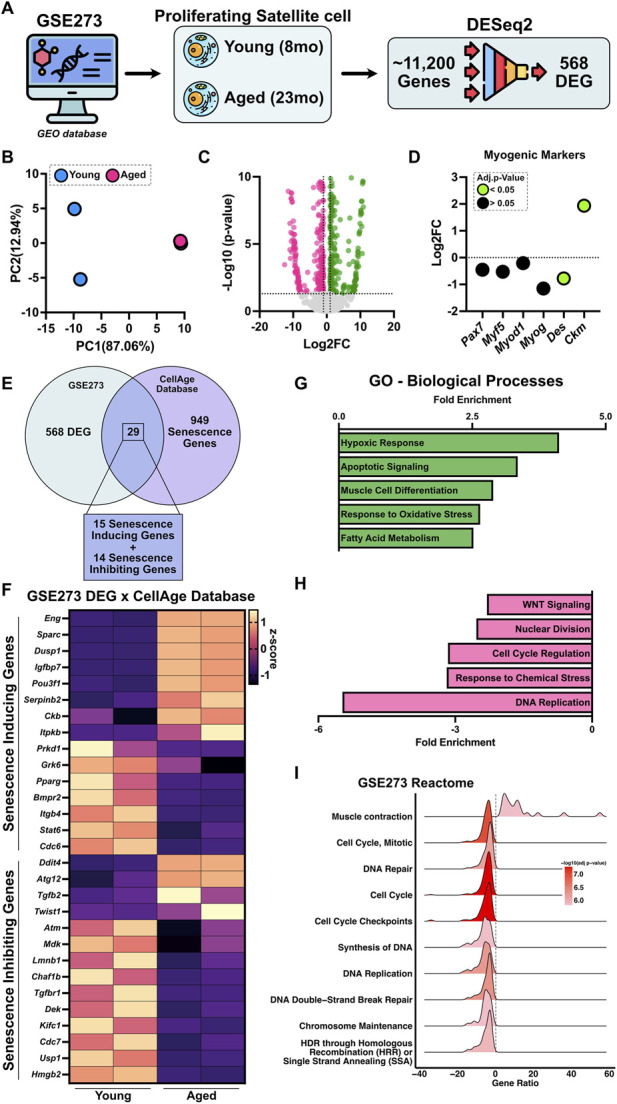
Aging induces transcriptional remodeling and senescence-related changes in proliferating satellite cells. **(A)** Schematic overview of the analysis pipeline for dataset GSE273, which profiled isolated proliferating satellite cells from young (8-month-old) and aged (23-month-old) mice. Differential expression analysis using DESeq2 identified 568 significantly altered genes between groups. **(B)** Principal component analysis (PCA) of variance-stabilized gene expression data shows clear separation between young and aged cells, with PC1 and PC2 explaining 87.06% and 12.94% of the total variance, respectively. **(C)** Volcano plot depicting differentially expressed genes (upregulated in green and downregulated in pink). **(D)** Expression profiles of canonical myogenic markers (*Pax7*, *Myf5*, *Myod1*, *Myog*, *Des*, and *Ckm*) between aged and young proliferating satellite cells. Data are shown as Log2FC (aged vs. young), where positive values indicate higher expression in aged cells. Green points denote genes with an adjusted p < 0.05, while black points represent non-significant changes. **(E)** Venn diagram showing the overlap between GSE273 DEGs and the CellAge Build 3 senescence gene database, identifying 29 senescence-associated genes. **(F)** Heatmap displaying the expression patterns of the 29 overlapping senescence-inducing and -inhibiting genes. **(G)** Gene ontology (GO) enrichment analysis of biological processes reveals upregulation (green) of stress-responsive and differentiation pathways, and **(H)** downregulation (pink) of pathways related to cell cycle, DNA replication, and WNT signaling. **(I)** Reactome pathway analysis highlights increased enrichment of skeletal muscle contraction-related pathways and reduced representation of genomic maintenance processes, including DNA repair, replication, and chromatin organization, in aged proliferating satellite cells.

To examine how aging impacts proliferating satellite cells, we analyzed dataset GSE273, which profiled proliferating satellite cells isolated using a pre-plate method from young (8-month-old) and aged (23-month-old) mice (Figure 3A). Of the 11,240 genes analyzed, 568 were identified as differentially expressed using DESeq2 (Figure 3A), indicating a distinct aging-associated transcriptional signature. PCA of variance-stabilized data revealed strong separation between young and aged cells, with PC1 and PC2 accounting for 87.06% and 12.94% of the variance, respectively (Figure 3B). Differential expression analysis identified 295 upregulated and 273 downregulated genes in aged cells compared to young proliferating satellite cells (Figure 3C). Analysis of canonical myogenic markers showed that *Des* expression was reduced, while *Ckm* was elevated in aged proliferating cells, suggesting a shift toward structural and metabolic maturation (Figure 3D). Overlap with the CellAge Build 3 database revealed 29 senescence-associated genes including 15 inducers and 14 inhibitors (Figures 3E,F). Of the inducing genes, 8 were upregulated (*Eng*, *Sparc*, *Dusp1*, *Ifgbp7*, *Pou3f1*, *Serpinb2*, *Ckb* and *Itpkb*) and 7 downregulated in aged cells (*Prkd1*, *Grk6*, *Pparg*, *Bmpr2*, *Itgb4*, *Stat6*, *Cdc6*), while 4 senescence inhibiting genes were upregulated (*Ddit4*, *Atg12*, *Tgfb2*, *Twist1*) and 10 were downregulated (*Atm*, *Mdk*, *Lmnb1*, *Chaf1b*, *Tgfbr1*, *Dek*, *Kifc1*, *Cdc7*, *Usp1*, *Hmgb2*). This combination of increased pro-senescent and reduced anti-senescent signals suggests that aging disrupts the balance of senescence regulation in satellite cells. GO analysis revealed enrichment of pathways associated with hypoxic response, apoptotic signaling, muscle differentiation, oxidative stress, and fatty acid metabolism (Figure 3G), suggesting a shift toward a more metabolically active and stress-responsive phenotype even in the absence of activation. In contrast, key processes involved in DNA replication, cell cycle regulation, nuclear division, response to chemical stress, and WNT signaling were significantly downregulated (Figure 3H). To further explore these functional differences, we performed pathway enrichment analysis using the Reactome pathway database. Consistent with the GO results, aged satellite cells exhibited increased expression of genes related to muscle contraction (Figure 3I), reinforcing the possibility of premature or inappropriate activation of myogenic programs. In contrast, pathways associated with DNA repair, cell cycle checkpoints, DNA replication, and chromatin maintenance were downregulated (Figure 3I), highlighting a loss of genomic stability and proliferative competence in aged cells. These transcriptional changes suggest that aged proliferating satellite cells may be primed for differentiation at the expense of self-renewal and genome integrity, contributing to their functional exhaustion with age.

**FIGURE 4:**
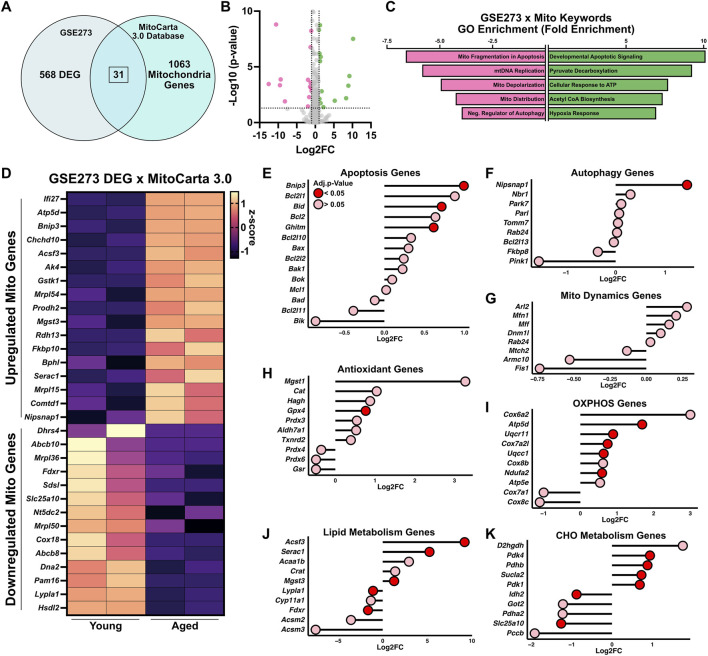
Aging alters mitochondrial gene expression and metabolic stress pathways in proliferating satellite cells. **(A)** Differentially expressed genes (DEGs) from GSE273 were cross-referenced with the MitoCarta 3.0 database to identify 31 mitochondrial-associated DEGs in aged proliferating satellite cells. **(B)** Volcano plot highlighting mitochondrial DEGs (upregulated in green and downregulated in pink). **(C)** Gene ontology (GO) enrichment analysis of mitochondrial DEGs shows distinct pathway enrichment among upregulated (green) and downregulated (pink) genes. **(D)** Heatmap displaying expression patterns of the 31 mitochondrial DEGs. Lollipop plots of selected mitochondrial-related genes, categorized into seven functional categories: **(E)** apoptosis, **(F)** autophagy, **(G)** antioxidant, **(H)** mitochondrial dynamics, **(I)** oxidative phosphorylation (OXPHOS), **(J)** lipid metabolism, and **(K)** carbohydrate (CHO) metabolism.

To investigate whether mitochondrial programs are altered in aged proliferating satellite cells, the 568 DEGs were cross-referenced with the MitoCarta 3.0 database (Figure 4A). This analysis identified 31 overlapping mitochondrial-associated genes, including 17 upregulated and 14 downregulated transcripts in aged compared to young satellite cells (Figures 4A,B). GO analysis of the upregulated mitochondrial genes revealed enrichment in pathways related to developmental apoptotic signaling, pyruvate decarboxylation, cellular response to ATP, acetyl-CoA biosynthesis, and hypoxia response (Figure 4C), suggesting a shift in mitochondrial function toward stress adaptation and altered metabolic signaling. In contrast, the downregulated mitochondrial genes were associated with critical processes including mitochondrial fragmentation, mitochondrial DNA replication, mitochondrial membrane depolarization and distribution, as well as negative regulators of autophagy, indicating impairments in mitochondrial quality control (Figure 4C).

To further explore the biological relevance of the transcriptomic changes, all genes overlapping with the MitoCarta database were categorized into one of seven mitochondrial and stress-related functional categories, as previously mentioned. In the apoptosis and autophagy categories, aged satellite cells showed upregulation of *Bnip3* (Log2FC = 1), *Bid* (Log2FC = 0.71), *Ghitm* (Log2FC = 0.61), and *Nipsnap1* (Log2FC = 1.4), suggesting increased apoptotic priming and altered mitophagy potential (Figures 4D–F). No genes related to mitochondrial dynamics were significantly altered (Figure 4G), indicating that transcriptional regulation of dynamics may be minimal at this stage. In the antioxidant gene category, *Gpx4* was upregulated (Log2FC = 0.76) (Figure 4H). In the OXPHOS category, several mitochondrial respiratory chain components were upregulated in aged cells, including *Atp5d* (Log2FC = 1.7), *Uqcr11* (Log2FC = 0.9), *Cox7a2l* (Log2FC = 0.74), *Uqcc1* (Log2FC = 0.63), and *Ndufa2* (Log2FC = 0.59) (Figure 4I), consistent with enhanced mitochondrial energy demands or remodeling. For lipid metabolism, aged cells exhibited upregulation of *Acsf3* (Log2FC = 9.2), *Serac1* (Log2FC = 5.2), and *Mgst3* (Log2FC = 1.27), while *Fdxr* (Log2FC = −1.65) and *Lypla1* (Log2FC = −1) were downregulated (Figure 4J), suggesting shifts in lipid synthesis, transport, and redox balance. Finally, in the carbohydrate metabolism category, genes such as *Pdk4* (Log2FC = 0.94), *Pdk1* (Log2FC = 0.68), *Pdhb* (Log2FC = 0.88), and *Sucla2* (Log2FC = 0.73) were upregulated, while *Idh2* (Log2FC = −0.87) and *Slc25a10* (Log2FC = −1.3) were downregulated. These changes indicate a rewiring of glucose oxidation and TCA cycle activity in aged satellite cells. Together, these coordinated transcriptional changes across multiple mitochondrial and metabolic pathways underscore the extent to which aging alters the energetic and stress response landscape of proliferating satellite cells.

### Early differentiation of satellite cells following BaCl_2_ injury reveals age-dependent dysregulation of senescence and mitochondrial programs

**FIGURE 5:**
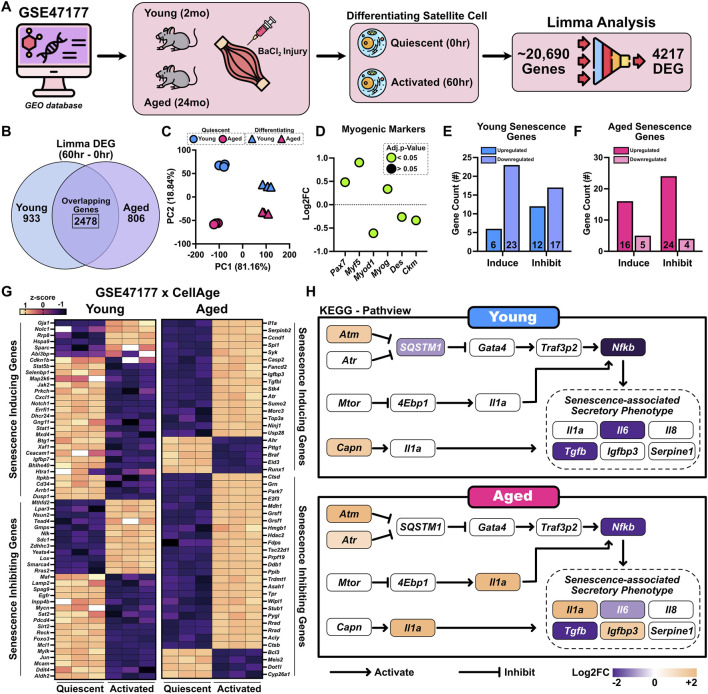
Activation induces age-dependent transcriptional remodeling and SASP engagement in differentiating satellite cells. **(A)** Schematic overview of the analysis pipeline for dataset GSE47177, which profiled satellite cells isolated from young (2-month-old) and aged (24-month-old) mice under resting conditions (0 h) or 60 h after BaCl_2_-induced skeletal muscle injury. Differential expression analysis using limma identified 4,217 significantly altered genes. **(B)** Venn diagram showing overlapping and age-specific DEGs following activation: 2,478 genes were shared, 933 were unique to young, and 806 were unique to aged satellite cells. **(C)** Principal component analysis (PCA) illustrating separation by age and activation state (quiescent cells shown as circles; differentiating cells at 60 h shown as triangles; young in blue, aged in red). **(D)** Expression profiles of canonical myogenic markers (*Pax7*, *Myf5*, *Myod1*, *Myog*, *Des*, and *Ckm*) in early differentiating satellite cells (60 h post-injury) from aged and young mice. Data are shown as Log2FC (aged vs. young), where positive values indicate higher expression in aged cells. Green points denote genes with an adjusted p < 0.05, while black points represent non-significant changes. **(E,F)** CellAge analysis reveals distinct patterns of senescence-associated gene regulation in young and aged activated satellite cells. **(G)** Heatmap of differentially expressed senescence-related genes. **(H)** Pathview diagram showing expression of SASP components, including upregulation of *Il1a* and *Igfbp3* in aged cells.

Our analysis thus far focused on quiescent and proliferating satellite cells, where aging already induces distinct transcriptional alterations. However, satellite cells undergo extensive remodeling upon differentiation stimuli (Sin et al., 2016; Baechler et al., 2019; Rahman and Quadrilatero, 2023; 2021; Rahman et al., 2025a), and we next sought to understand how aging influences this transition and whether mitochondrial or senescence-related programs are further impacted. To investigate this, we analyzed dataset GSE47177, which compared satellite cells from young and aged mice 60 h after BaCl_2_ induced injury, a time point that reflects early differentiation of satellite cells (Borok et al., 2021). Differential gene expression analysis was conducted between early differentiating (60 h) and quiescent (0 h; uninjured) satellite cells within each age group. Of the 20,690 genes analyzed, 4,217 genes were differentially expressed (Figure 5A). Among these, 2,478 DEGs were shared between young and aged satellite cells, while 933 DEGs were unique to young and 806 were unique to aged mice (Figure 5B). PCA showed clear separation between conditions, with PC1 explaining 81.16% and PC2 explaining 18.84% of the variance, confirming substantial transcriptional divergence during early differentiation, modulated by age (Figure 5C). Investigation of select myogenic markers revealed all were significantly altered, with aged cells showing higher *Pax7*, *Myf5*, and *Myog* but lower *Myod1*, *Des*, and *Ckm* expression (Figure 5D). To examine senescence-associated changes during early differentiation, DEGs were overlapped with the CellAge Build 3 database. In the young early differentiation response, there was a greater number of downregulated senescence-inducing and senescence-inhibiting genes (23 and 17, respectively) (Figure 5E). In contrast, the aged differentiation response showed upregulation of both senescence-inducing and senescence-inhibiting genes (16 and 24, respectively), suggesting potential dysregulation or compensatory signaling in aged satellite cells during regeneration (Figures 5F,G). To gain further mechanistic insight, we performed KEGG pathway analysis followed by Pathview visualization. This revealed selective activation of the SASP in aged differentiating satellite cells. Notably, *Il1a* and *Igfbp3*, both canonical SASP components, were upregulated in aged cells (Figure 5H). Interestingly, *Nfkb* and *Il6*, central regulators of SASP, showed lower expression in aged muscle, suggesting that SASP activation may be partially uncoupled from canonical inflammatory signaling or may represent a modified or attenuated SASP profile in aged satellite cells (Figure 5H).

**FIGURE 6:**
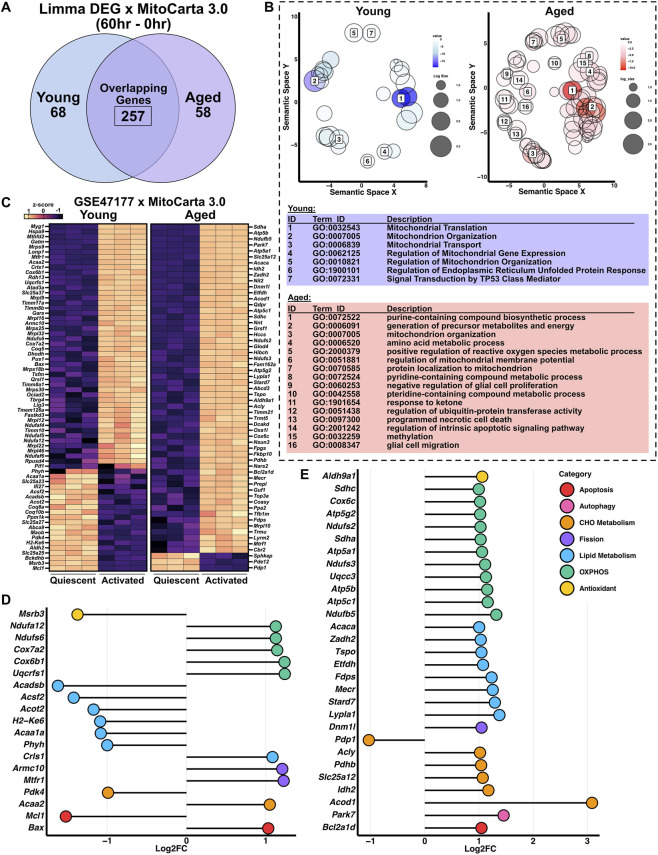
Mitochondrial gene remodeling during activation is enhanced and dysregulated in aged differentiating satellite cells. **(A)** Venn diagram of mitochondrial DEGs unique or shared between young and aged activation. **(B)** REVIGO-simplified GO terms of mitochondrial DEGs. Blue indicates GO terms enriched in young differentiating satellite cells, and pink indicates GO terms enriched in aged differentiating satellite cell. **(C)** Heatmap showing mitochondrial gene expression patterns. **(D,E)** Lollipop plots of mitochondrial genes categorized into functional categories for **(D)** young and **(E)** aged satellite cells.

To further examine mitochondrial remodeling during satellite cell differentiating and how it is influenced by aging, we cross-referenced differentially expressed genes from GSE47177 with the MitoCarta 3.0 database. This analysis identified 68 mitochondrial-associated DEGs unique to young mice, 58 unique to aged mice, and 257 shared between both groups, indicating a substantial reprogramming of mitochondrial gene expression during differentiation (Figure 6A). To better interpret the functional implications of these changes, we used REVIGO to semantically simplify enriched GO terms. In young differentiating satellite cells, the most prominent pathways involved mitochondrial translation, organization, transport, and regulation of mitochondrial gene expression, along with stress-related responses such as the unfolded protein response (UPR) and TP53 signaling (Figure 6B). In contrast, aged differentiating satellite cells showed enrichment of less clearly defined and more metabolically skewed pathways, including mitochondrial biosynthesis and stress-related signaling such as apoptosis (Figure 6B). Heatmap visualization revealed a greater number of upregulated mitochondrial genes in aged compared to young differentiating satellite cells (Figure 6C). Notably, young cells showed relatively few mitochondrial DEGs across the predefined functional categories of apoptosis, autophagy, dynamics, antioxidant, OXPHOS, lipid metabolism, and carbohydrate metabolism. These genes showed mixed changes in expression, with a modest increase in OXPHOS-related genes and a decrease in lipid metabolism genes (Figure 6D). A small subset of mitochondrial genes, including cytochrome c oxidase and ATP synthase subunits (Cox6 and Atp5 families), were shared across all aged satellite cell states, although the functional significance of this overlap remains unclear. We note that one limitation is that some functional categories contain only a few genes. These were retained to provide a complete overview of the mitochondrial pathways affected. Nonetheless, aged differentiating cells showed broader and more consistent upregulation across multiple mitochondrial categories, suggesting a more widespread and potentially dysregulated transcriptional response (Figure 6E).

## Discussion

Aging is known to impair the regenerative capacity of skeletal muscle, partially due to functional decline in satellite cells (Chakkalakal and Brack, 2012; Brack and Muñoz-Cánoves, 2016); however, the transcriptional basis of this decline remains poorly defined. In particular, age-related changes associated with mitochondrial remodeling and senescence have not been comprehensively characterized. In the present study, we performed a comparative transcriptomic analysis of quiescent, proliferating, and early differentiating satellite cells from young and aged mice using three publicly available datasets to investigate the effects of aging on mitochondrial and senescence-associated gene programs. Our findings reveal that: 1) aged satellite cells exhibit a dysregulated senescence profile; 2) aged satellite cells show a broader more disorganized differentiation response marked by inflammatory, apoptotic, and metabolic stress signaling, in contrast to the more focused transcriptional program seen in young cells; and 3) mitochondrial transcriptional changes are modest in quiescence and more prominent in proliferating and differentiating cells, particularly in aged cells, where they are linked to oxidative stress response, and apoptosis. Together, these data suggest that aging destabilizes the transcriptional networks governing stress resistance and metabolic remodeling in muscle stem cells, thereby compromising regenerative potential.

### Aged satellite cells exhibit complex and possibly compensatory senescence signatures

Aging is associated with a progressive decline in stem cell function, driven in part by senescence (Carlson and Faulkner, 1989; Conboy and Rando, 2005; Brack and Rando, 2007; Conboy et al., 2013; Sousa-Victor et al., 2014a; Brack and Muñoz-Cánoves, 2016). Senescent cells are classically defined as having dysregulated cell cycle function in response to stress and secrete inflammatory factors that disrupt the local microenvironment (He et al., 2021; Moiseeva et al., 2023; Wang et al., 2025). While this senescence response serves to prevent the expansion of damaged stem cells (McHugh and Gil, 2018), it can also impair tissue regeneration and contribute to age-related dysfunction (Brack and Muñoz-Cánoves, 2016). Across all datasets, aging altered the regulation of senescence-associated genes in satellite cells at every stage. Aged quiescent cells upregulated both pro- and anti-senescence genes, while proliferating cells showed a more balanced distribution of inducers and inhibitors. While senescence is classically defined by growth arrest, the co-expression of opposing regulators may suggest an unstable transcriptional state in aged satellite cells. This unstable balance may reflect attempts to resist against full senescence or could represent heterogeneity in the aged cell population. This idea is supported by studies showing that aged satellite cells often reside in a pre-senescent state, exhibiting features of senescence such as SASP signaling without fully committing to proliferative arrest (Chakkalakal et al., 2012; Chakkalakal and Brack, 2012; Bernet et al., 2014; Sousa-Victor et al., 2014a; Lazure et al., 2023). Nonetheless, bulk analysis may not capture cell-to-cell variation, as single-cell studies have revealed distinct satellite cell clusters with elevated stress and inflammatory signatures and progressive activation of stress-response pathways during differentiation (L’honoré et al., 2018; Pala et al., 2018). These findings highlight that population-level transcriptomics can mask functionally relevant heterogeneity in aged satellite cells.

Functionally, this dysregulated landscape could impair regenerative capacity by both intrinsic mechanisms and paracrine effects given that SASP-related cytokines secreted by senescent cells have been shown to impair niche integrity and stem cell function (He et al., 2021; Moiseeva et al., 2023; Wang et al., 2025). In GSE47177, aged satellite cells entering early differentiation upregulated both senescence-inducing and inhibitory genes, unlike young cells, which broadly downregulated these programs. This persistence of senescence-related activity following injury suggests delayed or incomplete suppression of stress signaling in aged muscle (Sousa-Victor et al., 2014a). Aged differentiating cells also displayed a broader and less coordinated transcriptional response, skewed toward inflammatory, apoptotic, and oxidative-stress pathways (Jejurikar et al., 2006; Lazure et al., 2023; Rahman et al., 2024). In contrast, young cells activated a focused program of OXPHOS, mitochondrial translation, and myogenic differentiation. Moreover, aged cells showed disproportionate upregulation of mitochondrial genes involved in lipid metabolism, biosynthesis, and apoptosis, suggesting a reactive mitochondrial transcriptome that may reflect attempts to compensate for accumulated dysfunction but also failure to complete the metabolic transition required for myogenic progression. Together, these findings indicate that aging promotes sustained senescence signaling and stress-biased mitochondrial remodeling across multiple activation states, likely contributing to impaired differentiation and a pro-inflammatory regenerative environment.

### Dysregulated differentiation and SASP signaling are characteristic of aged satellite cells

Our findings suggest that aging not only alters the baseline transcriptional state of satellite cells but also fundamentally reshapes their response to differentiation stimuli. This is consistent with previous work showing that aged satellite cells fail to properly engage the transcriptional programs required for efficient regeneration and instead activate stress and inflammatory responses (Sousa-Victor et al., 2014a; Lazure et al., 2023). A mild induction of SASP-related genes was also observed in young differentiating cells, likely reflecting a transient, physiological stress response that accompanies early myogenic remodeling. In contrast, aged cells exhibited a stronger and more sustained activation, suggesting failure to properly resolve this transient program, resulting in persistent inflammatory signaling and impaired differentiation. The broader activation of stress-related mitochondrial pathways in aged satellite cells could reflect both intrinsic dysfunction and maladaptive activation of compensatory mechanisms in an attempt to restore metabolic balance. This aligns with prior studies showing that aged satellite cells exhibit impaired activation and proliferative capacity following injury (Sousa-Victor et al., 2014b). Our results extend these findings by showing that aged satellite cells activate distinct gene programs that may interfere with successful progression through the regenerative process. Furthermore, aged differentiating satellite cells displayed upregulation of SASP components, including *Il1a* and *Igfbp3*, reinforcing the possibility that aged cells contribute to a pro-inflammatory or dysfunctional regenerative microenvironment. Mechanistically, IL1A can act in an autocrine and paracrine manner through IL1R1 to activate NFKB and MAPK signaling (Weber et al., 2010), thereby amplifying inflammatory gene expression and potentially sustaining a chronic stress environment that impairs differentiation. In parallel, *Igfbp3* reduces bioavailable IGF1 and limits downstream PI3K-AKT-MTOR signaling (Baxter, 2023), restricting anabolic and pro-differentiation pathways essential for effective myogenesis. These events may trap aged satellite cells in a state where inflammatory cues dominate over regenerative programs. This aligns with reports that aged muscle accumulates senescent cells expressing *Ccl2*, *Ccl7*, *Timp2*, and *Igfbp4* (Moiseeva et al., 2023). Interestingly, key SASP regulators such as *Nfkb* and *Il6* were not similarly elevated, suggesting that SASP signaling in aged cells may be rewired or attenuated compared with canonical profiles. Whether this reflects a compensatory adaptation or an inability to resolve inflammation remains unclear.

We also observed transcriptional upregulation of mitophagy-related genes such as *Bnip3* and *Nipsnap1* in both quiescent and proliferating cells, as well as *Park7* during differentiation, potentially reflecting increased mitochondrial stress and the need for organelle clearance. Previous work has demonstrated that mitophagy is essential for preserving stemness, and that mitochondrial dysfunction in aged satellite cells contributes to senescence and regenerative failure (García-Prat et al., 2016). Mitophagy is also dynamically engaged during myogenic differentiation, where it facilitates mitochondrial remodeling to meet the metabolic demands of lineage progression (Sin et al., 2016; Baechler et al., 2019; Rahman et al., 2025a). However, our analysis captures only transcriptional changes that may not necessarily indicate increased functional mitophagic flux given that protein translocation to the mitochondria, interaction with autophagic machinery, and delivery to the lysosome can all limit effective mitochondrial clearance despite elevated gene expression. Nonetheless, mitochondrial dysfunction can reinforce senescence via ROS production and impaired ATP output (Passos et al., 2010), and the upregulation of *Il1a* and *Igfbp3* in aged differentiating cells suggests that mitochondrial stress is directly linked to inflammatory signaling. *Il1a* activates inflammatory pathways to sustain pro-senescent paracrine signaling, while *Igfbp3* suppresses growth signals, limiting mitochondrial biogenesis and differentiation. These factors could create a feed-forward loop where SASP activity and impaired mitochondrial remodeling jointly sustain senescence and blunt regeneration. The interplay between mitophagy and senescence thus represents a potential mechanism of regenerative failure in aged muscle that warrants further investigation.

### Mitochondrial and metabolic transcriptional remodeling occurs in both quiescent and proliferative states, but is more pronounced during differentiation

The metabolic profile of stem cells is important in maintaining quiescence and its changes are needed to drive terminal differentiation (Vertel and Fischman, 1977; Barbieri et al., 2011; Latil et al., 2012; Robinson et al., 2019; Rahman and Quadrilatero, 2023). Our analyses show that mitochondrial transcriptional remodeling is evident in aged quiescent and proliferating satellite cells and becomes more pronounced during differentiation. In GSE273 and GSE50821, only a small subset of DEGs overlapped with MitoCarta, primarily involving fatty acid metabolism and apoptotic signaling. Aged quiescent cells upregulated genes related to fatty acid and ketone metabolism (*Bdh1*, *Oxct1*, *Acaa2*), as well as peroxisomal lipid oxidation (*Acot3*, *Cyp27a1*, *Phyh*), suggesting a shift toward oxidative lipid metabolism and enhanced redox regulation. This may reflect age-related metabolic reprogramming driven by mitochondrial stress, altered nutrient sensing, or lipid availability in the aged niche. Aged proliferating cells also displayed transcriptional signs of metabolic inefficiency. Upregulation of *Pdhb* along with its inhibitors *Pdk1* and *Pdk4*, and increased *Sucla2* expression, suggest disrupted coordination between glycolysis and the TCA cycle. In parallel, reduced *Idh2* and *Slc25a10* expression indicate diminished mitochondrial NADPH generation and substrate transport. Together, these changes imply that aged cells may not efficiently reprogram toward the glycolytic metabolism that normally supports satellite cell activation, reflecting metabolic inflexibility with age.

Following differentiation stimuli (GSE47177), mitochondrial DEGs increased markedly, with enrichment in translation, OXPHOS, transport, and stress-response pathways. This metabolic shift toward higher OXPHOS supports myogenesis (Ryall et al., 2015), yet aged cells exhibited a less coordinated pattern dominated by stress and apoptotic pathways. The pre-existing lipid oxidation bias in aged cells may limit metabolic flexibility, forcing differentiation from a non-optimal energetic state. This likely contributes to impaired engagement of OXPHOS and activation of oxidative stress and apoptosis pathways, thereby compromising regeneration. Furthermore, differentiating aged cells also showed enrichment of biosynthetic and apoptotic pathways, indicating disorganized or stress-reactive mitochondrial remodeling. This agrees with our previous findings that high-passage C2C12 myoblasts exhibit increased mitochondrial apoptotic signaling and impaired differentiation (Rahman et al., 2024). Consistent with the increase in mitochondrial apoptotic signaling, we observed upregulation of dual apoptotic and mitophagy molecule *Bnip3* in aged quiescent and proliferating satellite cells. *Bnip3* is also among the overlapping genes in response to injury that is conserved between young and aged satellite cells. It is noteworthy that *Bnip3* consistently emerges in our analyses. Our group has shown that *Bnip3* expression increases at the onset of skeletal muscle differentiation, and its importance is underscored by findings that C2C12 myoblasts lacking *Bnip3* fail to differentiate (Baechler et al., 2019; Rahman et al., 2025a). Furthermore, *Bnip3* has also appeared in prior transcriptomic studies of postnatal skeletal muscle development, and its protein levels are modulated by disuse in mature skeletal muscle (Rahman et al., 2025b; Rahman et al., 2025c). BNIP3 contains a BH3 domain that primes apoptosis and an LC3-interacting region that mediates mitophagy, making it an important regulator of cell fate and mitochondrial quality control. Thus, its upregulation in aged cells may reflect a chronic mitochondrial stress response, where apoptotic priming and selective mitophagy are simultaneously engaged but incompletely resolved. In this context, dysregulated BNIP3 signaling may represent a maladaptive attempt to remodel mitochondria during differentiation, contributing to the impaired regenerative capacity of aged muscle. However, more work is needed to fully elucidate this phenomenon. Nonetheless, other mitophagy-related genes, including *Pink1*, and the antioxidant chaperone *Park7*, were also elevated in aged differentiating cells, possibly representing compensatory activation of stress-response pathways aimed at stabilizing mitochondrial function rather than full organelle turnover. Such incomplete or maladaptive remodeling may allow persistence of dysfunctional mitochondria, further limiting regenerative efficiency in aged skeletal muscle.

## Conclusion

Our analysis reveals that aging fundamentally alters the transcriptional landscape of satellite cells, affecting both their resting state and response to injury. By comparing gene expression profiles across quiescent, proliferating, and early differentiating states, we found two major themes: disrupted regulation of senescence pathways and impaired mitochondrial remodeling. Aged satellite cells exhibited transcriptional changes in several senescence-associated genes, including both inducers and inhibitors of the pathway which may reflect heterogeneous activation of senescence-related programs within the aged satellite cell population. These signatures were also detectable during early differentiation, suggesting that senescence-associated gene activity persists and may contribute to an altered cellular environment that limits their regenerative potential. Mitochondrial gene expression was relatively stable in quiescent cells but became increasingly dysregulated with activation. Aged cells showed signs of metabolic stress and inefficiency, with broader and less coordinated transcriptional responses compared to young cells. During early differentiation, mitochondrial remodeling appeared disorganized and incomplete in aged cells, which may reflect poor adaptation to the bioenergetic demands of regeneration. Moreover, aging shifts satellite cells from a focused regenerative program to one dominated by stress, inflammation, and metabolic inflexibility, contributing to impaired muscle repair. These findings indicate that aging affects not only activation capacity but also broader gene networks essential for regeneration. Targeting mitochondrial and senescence-associated pathways may help preserve skeletal muscle function with age.

## Data Availability

The raw data supporting the conclusions of this article will be made available by the authors, without undue reservation.
